# A43 TOPICAL HEMOSTATIC AGENTS: A POTENTIAL BREAKTHROUGH IN MALIGNANCY-RELATED GI BLEEDING? A SYSTEMATIC REVIEW AND META-ANALYSIS OF RANDOMIZED CONTROLLED TRIALS

**DOI:** 10.1093/jcag/gwae059.043

**Published:** 2025-02-10

**Authors:** G S Minhas, A Bukhari, Y Yuan, G Leontiadis, L Laine, A Barkun, F Tse

**Affiliations:** Medicine, McMaster University, Hamilton, ON, Canada; Medicine, McMaster University, Hamilton, ON, Canada; Western University, London, ON, Canada; Medicine, McMaster University, Hamilton, ON, Canada; Yale University School of Medicine, New Haven, CT; McGill University, Montreal, QC, Canada; Medicine, McMaster University, Hamilton, ON, Canada

## Abstract

**Background:**

Malignancy-related gastrointestinal bleeding (GIB) has been challenging to treat with conventional endoscopic techniques. Topical hemostatic agents (THAs), which do not cause mucosal injury and can be applied over large surface areas, may offer a promising solution in cancer-related GIB.

**Aims:**

To assess the effectiveness and safety of THAs in malignancy-related GIB compared to conventional endoscopic techniques.

**Methods:**

We conducted a systematic review using MEDLINE, EMBASE, and Cochrane Central Register of Controlled Trials until March 2024. RCTs that compared THA vs conventional endoscopic techniques in malignant GIB were included. Two authors conducted study selection, data extraction and quality assessment independently. The primary outcome was 30-day further bleeding. Further bleeding is a composite outcome of failure to achieve immediate hemostasis and rebleeding. Secondary outcomes included failure to achieve immediate hemostasis, 6-month mortality, 30-day rebleeding, blood transfusions needed, length of hospitalization, and adverse events. RevMan 5.4 was used to calculate pooled risk ratios (RR) with 95% confidence intervals (CI, random effects model). Heterogeneity was assessed by Chi 2 (P<0.15) and I 2 tests (>25%). We assessed the certainty of evidence (CoE) for each outcome using the GRADE approach.

**Results:**

Results: We identified 5100 citations and 4 RCTs with 122 patients were included in the analysis. There was no significant difference between THA and conventional endoscopic methods with respect to 30-day further bleeding through THAs reduce the risk of immediate hemostatic failure. There was no significant difference noted in 30-day rebleeding. THAs did not significantly impact 6-month mortality or blood transfusions. THAs were found to increase the average length of hospitalization by 4.28 days.

**Conclusions:**

TC-325 appears superior to conventional endoscopic techniques in achieving immediate hemostasis for malignancy-related

GIB. It may reduce further bleeding over 30 days as well, but the evidence is very uncertain and further research is needed to assess bleeding risk over this period and beyond. TC-325 did not appear to reduce 6-month mortality.

Table 1. Summary of Findings

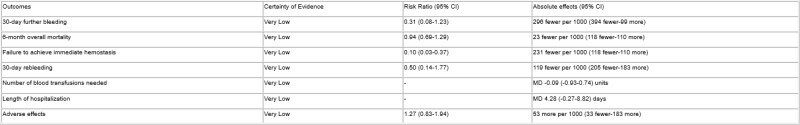

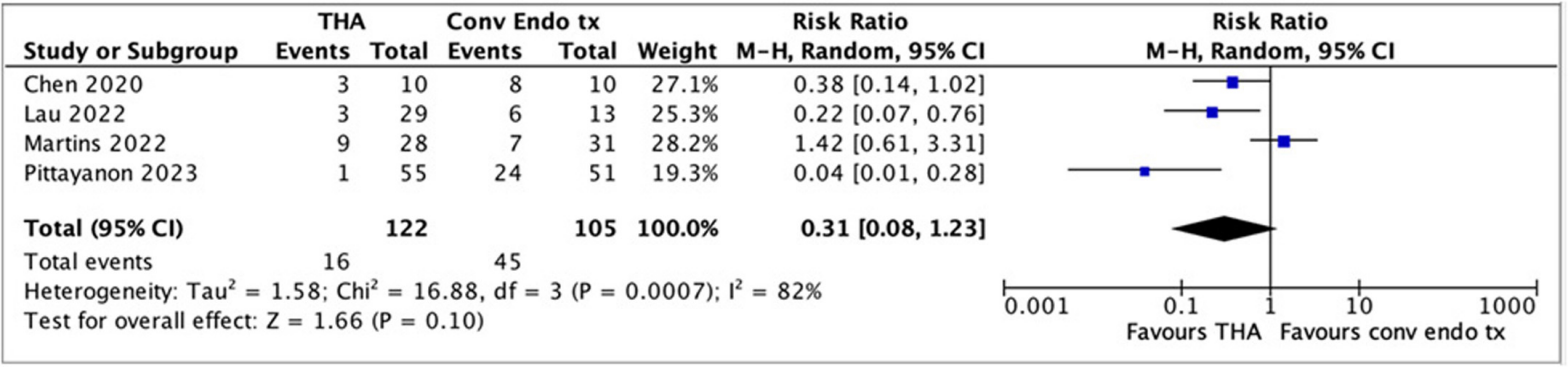

Forest plot of further bleeding

**Funding Agencies:**

